# Relationship between seasons and pregnancy rates during intrauterine insemination. A historical cohort

**DOI:** 10.1590/1516-3180.2018.05111220719

**Published:** 2019-10-31

**Authors:** Meryem Kuru Pekcan, Esma Sarıkaya, Aytekin Tokmak, Hasan Ali İnal, Nafiye Yılmaz

**Affiliations:** I MD. Specialist Doctor, Department of Obstetrics and Gynecology, Dr. Zekai Tahir Burak Women’s Health Education and Research Hospital, Ankara, Turkey.; II MD. Professor, Department of Obstetrics and Gynecology, Dr. Zekai Tahir Burak Women’s Health Education and Research Hospital, Ankara, Turkey.; III MD. Associate Professor, Department of Obstetrics and Gynecology, Dr. Zekai Tahir Burak Women’s Health Education and Research Hospital, Ankara, Turkey.; IV MD. Associate Professor, Department of Obstetrics and Gynecology, Dr. Zekai Tahir Burak Women’s Health Education and Research Hospital, Ankara, Turkey.; V MD. Professor, Department of Obstetrics and Gynecology, Dr. Zekai Tahir Burak Women’s Health Education and Research Hospital, Ankara, Turkey.

**Keywords:** Seasons, Circadian rhythm, Ovulation induction, Pregnancy, Melatonin

## Abstract

**BACKGROUND:**

The underlying cause of seasonal infertility in humans is unclear, but is likely to be ­multifactorial.

**OBJECTIVE:**

The aim of our study was to compare the pregnancy rates among infertile women who underwent induced ovulation and intrauterine insemination (IUI) with the season in which the fertility treatment was performed.

**DESIGN AND SETTING:**

This retrospective cohort study was conducted on 466 patients who were treated in the reproductive endocrinology and infertility outpatient clinic of a tertiary-level women’s healthcare and maternity hospital.

**METHODS:**

Retrospective demographic, hormonal and ultrasonographic data were obtained from the patients’ medical records. Clomiphene citrate or gonadotropin medications were used for induced ovulation. The patients were divided into four groups according to the season (spring, winter, autumn and summer) in which fertility treatment was received. Clinical pregnancy rates were calculated and compared between these four groups.

**RESULTS:**

There were no significant differences between the seasonal groups in terms of age, infertility type, ovarian reserve tests, duration of infertility, medications used or length of stimulation. A total of 337 patients (72.3%) were treated with clomiphene citrate and 129 (27.7%) with gonadotropin; no significant difference between these two groups was observed. The clinical pregnancy rates for the spring, winter, autumn and summer groups were 15.6% (n = 24), 8.6% (n = 9), 11.5% (n = 13) and 7.4% (n = 7), respectively (P = 0.174).

**CONCLUSIONS:**

Although the spring group had the highest pregnancy rate, the rates of successful IUI did not differ significantly between the seasonal groups.

## INTRODUCTION

Many environmental factors influence human fertility outcomes. Although extensive research has shown that mammalian fertility is influenced by seasonal changes, few studies have specifically evaluated seasonal effects on the human reproductive system.^1^ Seasonal infertility may be due to physiological changes that are season-dependent. It was previously shown that season-dependent high environmental temperatures negatively affect sexual function and nutrient intake.^2^^,^^3^^,^^4^ Conversely, a five-year study conducted in France, in which the effect of the photoperiod on seasonal infertility was examined, demonstrated that seasonal infertility was independent of environmental temperatures.^5^

Melatonin affects several daily and seasonal rhythms, such as endocrine signaling during both circadian time and daytime. Melatonin concentrations follow different circadian rhythm in different living things. Nighttime exposure to light inhibits melatonin synthesis and secretion in both animal models^6^ and humans.^7^ Serum levels of melatonin are affected by the complex interaction of daily rhythm, exogenous factors and endogenous factors. Melatonin receptors have been identified in human reproductive tissues.^8^

Melatonin plays a major role in reproductive activity and blastocyst implantation. It is transferred maternally to the fetus via the placenta or milk, during pregnancy and lactation, respectively, thus indicating that the maternal photoperiod is transferred to the fetus.^9^ In addition, it has been shown that melatonin causes seasonal changes relating to fertilization, embryo quality, sperm concentration and chromatin condensation rates.^10^ The impact of melatonin on reproductive function in women has been demonstrated in studies that showed that high levels of melatonin cause amenorrhea, which leads to decreased secretion of gonadotropin and prolactin in response to the photoperiod.^11^

Several retrospective studies have evaluated the impact of seasonal variation on in vitro fertilization outcomes. Some of these studies considered climatic conditions, especially temperature and the number of hours of daylight.^12^ However, few studies have evaluated seasonal effects on pregnancy rates among human patients undergoing intrauterine insemination (IUI).^13^^,^^14^ Seasonality of infertility treatment may alter reproductive performance; and therefore, the timing of infertility treatment may result in improved pregnancy rates.

## OBJECTIVE

This study compared pregnancy rates during different seasons in which fertility treatments were performed, in order to investigate whether seasonal variations were associated with pregnancy outcomes among infertile women who underwent induced ovulation and IUI.

## METHODS

### Study design, settings and ethics

A retrospective cohort study based on medical records was conducted among infertile women who underwent induced ovulation with IUI treatment at Dr. Zekai Tahir Burak Women’s Health Education and Research Hospital between May 2013 and June 2015. Informed consent was not obtained from the participants because of the retrospective study design. The study was conducted in accordance with the principles of the Declaration of Helsinki and was approved by this hospital’s Institutional Review Board (date: May 27, 2015; decision no. 19).

All consecutive infertile couples who met the inclusion criteria during the study period of two years were recruited for this study. All the study participants met the following criteria: < 41 years of age; normal hysterosalpingography (HSG) and/or laparoscopy findings; regular menstrual cycles with normal baseline levels of serum follicle-stimulating hormone (FSH), luteinizing hormone (LH), estradiol (E2), prolactin and thyroid-stimulating hormone (TSH); normal spermiogram parameters in the husband (according to the World Health Organization’s 2010 criteria);^15^ and serum progesterone levels > 5 ng/ml during the midluteal phase. Couples with poor ovarian reserve, endometriosis, tubal or uterine factors, male infertility or systemic disorders such as thyroid disease or diabetes mellitus were excluded from the study.

Patients undergoing treatments (both induced ovulation and IUI) that were performed in one season were included in the study. When a treatment (induced ovulation or IUI) overlapped with the previous or next season, the patient was excluded from the study.

This study was conducted in Ankara (latitude: 32.87° N, longitude: 39.87° E; altitude: 891 m). Ankara has a continental climate, which means that it has cold, snowy winters and hot, dry summers. Rainfall is mostly seen during the spring and autumn months. During the 24-month study period, the average seasonal air temperatures were 22.3 °C in the summer, 12.9 °C in the autumn, 1.4 °C in the winter, and 11.0 °C in the spring.^16^

Demographic, hormonal and ultrasonographic data relating to the patients were copied from the patients’ files and the hospital’s electronic database. The patients were divided into spring, winter, autumn and summer groups.

### Fertility treatments

Patients with unexplained or anovulatory infertility were first treated with clomiphene citrate. Women who received clomiphene citrate treatment but were unable to conceive were next treated with gonadotropin. Patients who declined clomiphene citrate treatment, were over the age of 35 years or had experienced infertility over a relatively long period of time (greater than or equal to three years) directly received gonadotropin treatment as the initial treatment. Induced ovulation was planned together with administration of clomiphene citrate (50-150 ­mg/­day, orally) or gonadotropin (37.5-150 IU of pure FSH or human menopausal gonadotropin, HMG), starting on day 2 or day 3 of the menstrual cycle.

Follicular development was monitored by means of transvaginal ultrasound on days 2-3 (baseline) and then on either day 8 of the gonadotropin cycle or day 12 of the clomiphene citrate cycle. Subsequent monitoring was performed until the follicular diameter reached 18 to 20 mm, and then a 250-mcg dose of recombinant human chorionic gonadotropin (rHCG) was administered subcutaneously.

The patients underwent a serum pregnancy test on day 14 following IUI. Clinical pregnancy was defined as the presence of a gestational sac with an accompanying fetal heartbeat detected by means of ultrasound, at least five weeks after IUI.

### Statistical analysis

All analyses were performed using the Statistical Package for the Social Sciences 15.0 (SPSS, Chicago, IL, USA) for Windows. Normal distribution of data was assessed using the Kolmogorov-Smirnov test. Continuous variables were presented as mean ± standard deviation (SD). Intragroup differences were investigated using one-way analysis of variance (ANOVA). Categorical variables were expressed as the number (with percentage). Differences between data categories were evaluated using the chi-square or Fisher’s exact test. Statistical significance was assumed based on a probability of 0.05.

The sample size calculation was performed using the DSS statistical software package for research sample size calculations.^17^ The primary aim of this study was to compare the differences in clinical pregnancy rate between the seasons. It was calculated that a minimum of 80 participants in each group would be required to demonstrate a difference of at least 10% between the groups, with a power of 80% at the 5% significance level. This difference of 10% was taken both from a pilot study^14^ and from our clinical experiments.

## RESULTS

A total of 466 women were enrolled in the study. The study participants were divided into four groups according to the season in which induced ovulation and IUI treatment were received. The spring, winter, autumn and summer groups contained 154, 105, 113 and 94 patients, respectively. No significant differences were observed between the groups in terms of age, primary infertility rate, baseline hormone levels, antral follicle count or duration of infertility (P > 0.05). The cycle characteristics, including the induced ovulation treatment protocol and duration of stimulation, were also similar among the groups (Table 1).

**Table 1.** Baseline, laboratory and clinical parameters of all seasonal groups

Season		Spring group (n = 154)	Winter group (n = 105)	Autumn group (n = 113)	Summer group ( n = 94)	P
Age (years)		27.18 ± 4.54	26.41 ± 4.88	26.97 ± 4.89	27.23 ± 5.17	0.575*
Basal FSH (IU/ml)		6.64 ± 1.86	6.69 ± 2.23	6.65 ± 2.01	6.33 ± 1.79	0.541*
Basal E_2_ (pg/ml)		41.78 ± 12.99	39.79 ± 15.47	39.91 ± 20.58	43.31 ± 18.55	0.747*
Duration of infertility (years)		4.02 ± 2.74	3.51 ± 2.19	3.49 ±1.80	3.32 ± 1.94	0.078*
Antral follicle count		9.41 ± 2.51	10.32 ± 5.18	9.53 ± 2.36	9.85 ± 2.20	0.301*
Duration of stimulation (days)		12.23 ± 1.55	12.10 ± 1.34	12.18 ± 1.33	12.03 ± 1.37	0.707*
Stimulation protocol	CC (%)	105 (68.2)	77 (73.3)	84 (74.3)	71 (75.5)	0.559**
	Gonadotropin (%)	49 (31.8)	28 (26.7)	29 (25.7)	23 (24.5)	
Peak E_2_ (pg/ml)		699.37 ± 556.73	740.88 ± 564.30	924.29 ± 712.02	629.09 ± 432.74	0.274*
Endometrial thickness on HCG day (mm)		8.46 ± 1.06	8.88 ± 1.74	8.44 ± 2.09	8.78 ± 1.99	0.084*
Clinical pregnancy rate (%)		24 (15.6)	9 (8.6)	13 (11.5)	7 (7.4)	0.174***

FSH = follicle stimulating hormone; E_2_ = estradiol; CC = clomiphene citrate; HCG = human chorionic gonadotropin.

P-values of less than 0.05 were considered statistically significant.

*Analysis of variance (ANOVA); **Chi-square test; ***Fisher’s exact test.

A total of 337 patients were treated with clomiphene citrate and 129 patients were treated with gonadotropin. There was no significant difference among the initial indications for gonadotropin. The number of patients who received clomiphene citrate treatment was 105 in the spring (68.2%), 77 in the winter (73.3%), 84 in the autumn (74.3%) and 71 in the summer (75.5%). The number of patients who received gonadotropin treatment was 49 in the spring (31.8%), 28 in the winter (26.7%), 29 in the autumn (25.7%) and 23 in the summer (24.5%).

The peak E2 level was highest in the autumn (924.29 ± 712.02 pg/­ml) and was lowest in the summer (629.09 ± 432.74 pg/ml). The endometrial thickness on the HCG day and the duration of stimulation (days) were similar in the four seasons (P = 0.084).

The overall clinical pregnancy rate was 11.4% in this cohort. The pregnancy rates in the spring, winter, autumn and summer groups were 15.6%, 8.6%, 11.5% and 7.4%, respectively (P > 0.05). Although the clinical pregnancy rate was highest in the spring season, there was no significant difference between the seasons.

## DISCUSSION

In this study, we aimed to investigate the seasonal variations of IUI success. To the best of our knowledge, this was the first study to analyze the seasonal variations of pregnancy rates following IUI in Turkey. Women were evaluated over a period of more than 24 months in this study. Regardless of whether the analysis compared all couples, age, day-3 FSH, duration of infertility (years) or duration of stimulation (days), the endometrial thickness on the HCG day (in mm) was not altered by seasonal effects. Although the clinical pregnancy rate among infertile couples varied according to the season, especially in the spring (15.6%), no statistically significant differences between the seasons were observed.

The seasonal effect on mammalian reproduction has long been known. Several studies have shown that hot weather reduces sperm quality and fertility rates.^18^^,^^19^^,^^20^ These effects have been also associated with melatonin production and secretion.^7^ Melatonin concentration shows a distinctive circadian rhythm in all species. Daily changes in the synthesis of melatonin are regulated by the ambient light-dark cycle. Light provides strong synchronization of the rhythms of the suprachiasmatic nuclei.^21^

The field of metabolomics or metabolic profiling investigates the connections between melatonin, circadian rhythm, sleep and metabolism.^22^^,^^23^ Declines in reproductive capacity due to seasonal infertility have been observed in the United States^24^ and in Germany.^25^ Further studies with large numbers of participants are needed in order to evaluate the role of melatonin rhythm and amplitude in human metabolism, and such evaluations may reveal new perspectives regarding the physiological role of melatonin.

Seasonal infertility has been correlated with a number of environmental factors, including the photoperiod. Although the photoperiod plays a role in seasonal infertility, high temperatures may also cause direct or cumulative adverse effects on fertility.^5^ High temperatures above the thermo-neutral zone have been shown to reduce birth rates and delay the onset of puberty.^26^ It has been suggested that heat stress is a probable factor in the development of seasonal infertility^27^ and that this negatively affects embryo development.^28^ Heat shock proteins, which appear in response to heat stress,^29^ are found in the ovaries.^30^ It has been shown that hyperthermia affects developing oocytes.

In a study by Palacios, the pregnancy rate after artificial insemination of dairy sheep was significantly affected by seasonal meteorological variables.^31^ In that study, winter was the season with the lowest percentage for overall fertility (42.4%), and this was significantly different (P < 0.001) from spring (45.4%), summer (45.6%) and autumn (46.0%). Successful inseminations were performed at significantly lower maximum temperatures in the summer.

Santolaria et al. investigated the pregnancy rates among sheep at the same latitude (41° N) between July and October. They examined the period from 12 days before insemination to 14 days after insemination and found that the pregnancy rate was lower when the temperature was above 30 °C, over a two-day period before insemination.^32^

In another study, Hashem et al. detected the presence of relativity of the estrous phase. They found that efficient fertile mating was positively correlated with high temperature and a long photoperiod (summer season conditions), while it was negatively correlated with rainfall (winter season condition) in Egypt.^33^

However, we did not find any significant difference in relation to seasonal changes. On the other hand, we did not measure temperature, weather or daily exposure to light.

The relationship between birth, fertility and seasonal change is unclear. Roenneberg and Aschoff^34^ defined a circadian rhythm that changed over time for birth rates around the world. Pregnancy rates in relation to seasonal fecundability were also analyzed in their study. They hypothesized that the biological rhythm of conception is influenced by social or by environmental factors. Their conclusion was that although conception and birth rhythms vary from country to country, these rhythms have changed their characteristics recently, after having remained stable for more than a century.

The limiting feature of our study was that we did not know the exact meteorological data and temperatures. The sleep patterns and stress levels, which may have been affected by melatonin, were unknown. In addition, changes to people’s lifestyles are increasing, as an inevitable consequence of modern life. Today, people live in houses with a constant temperature, are exposed to artificial light instead of sunlight and come into less and less contact with the external environment. All of these factors may lead to lower but more stable reproductive performance, through elimination of the confounding effects of environmental conditions.

## CONCLUSION

Although we found a higher pregnancy rate among women undergoing infertility treatment during the spring season, we did not detect that seasonal variation had any statistically significant influence on the success of IUI. New studies with higher power may find a significant difference between the outcomes from infertility treatment and seasonal changes. In this regard, further large-scale studies are required, in order to better evaluate the effects of seasonal variability on pregnancy.
